# Efficacy and safety of fremanezumab in patients with episodic and chronic migraine with documented inadequate response to 2 to 4 classes of migraine preventive medications over 6 months of treatment in the phase 3b FOCUS study

**DOI:** 10.1186/s10194-021-01279-7

**Published:** 2021-07-10

**Authors:** Messoud Ashina, Joshua M. Cohen, Maja Galic, Verena Ramirez Campos, Steve Barash, Xiaoping Ning, Yoel Kessler, Lindsay Janka, Hans-Christoph Diener

**Affiliations:** 1 Department of Neurology, Danish Headache Center, Rigshospitalet Glostrup, University of Copenhagen, Valdemar Hansens Vej 5, DK-2600 Glostrup, Copenhagen, Denmark; 2 Teva Branded Pharmaceutical Products R&D, Inc., West Chester, PA USA; 3 Teva Pharmaceuticals, Amsterdam, The Netherlands; 4 Institute for Medical Informatics, Biometry and Epidemiology, Medical Faculty of the University Duisburg-Essen, Essen, Germany

**Keywords:** Migraine, Calcitonin gene-related peptide, CGRP, Long-term safety, Long-term efficacy

## Abstract

**Background:**

Fremanezumab, a fully humanized monoclonal antibody (IgG2Δa) selectively targets the calcitonin gene-related peptide and has proven efficacy for the preventive treatment of migraine. In this study, we evaluated the long-term efficacy, safety, and tolerability of monthly and quarterly fremanezumab.

**Methods:**

Episodic migraine and chronic migraine patients completing the 12-week double-blind period of the FOCUS trial entered the 12-week open-label extension and received 3 monthly doses of fremanezumab (225 mg). Changes from baseline in monthly migraine days, monthly headache days of at least moderate severity, days of acute headache medication use, days with photophobia/phonophobia, days with nausea or vomiting, disability scores, and proportion of patients achieving a ≥50% or  ≥75% reduction in monthly migraine days were evaluated.

**Results:**

Of the 807 patients who completed the 12-week double-blind treatment period and entered the open-label extension, 772 patients completed the study. In the placebo, quarterly fremanezumab, and monthly fremanezumab dosing regimens, respectively, patients had fewer average monthly migraine days (mean [standard deviation] change from baseline: − 4.7 [5.4]; − 5.1 [4.7]; − 5.5 [5.0]), monthly headache days of at least moderate severity (− 4.5 [5.0]; − 4.8 [4.5]; − 5.2 [4.9]), days per month of acute headache medication use (− 4.3 [5.2]; − 4.9 [4.6]; − 4.8 [4.9]), days with photophobia/phonophobia (− 3.1 [5.3]; − 3.4 [5.3]; − 4.0 [5.2]), and days with nausea or vomiting (− 2.3 [4.6]; − 3.1 [4.5]; − 3.0 [4.4]). During the 12-week open-label extension, 38%, 45%, and 46% of patients, respectively, achieved a ≥50% reduction and 16%, 15%, and 20%, respectively, achieved a ≥75% reduction in monthly migraine days. Disability scores were substantially improved in all 3 treatment groups. There were low rates of adverse events leading to discontinuation (<1%).

**Conclusion:**

Fremanezumab demonstrated sustained efficacy up to 6 months and was well tolerated in patients with episodic migraine or chronic migraine and documented inadequate response to multiple migraine preventive medication classes.

**Trial registration:**

ClinicalTrials.gov NCT03308968 (FOCUS).

## Introduction

The burden of migraine is substantial and it includes social and economic burdens in addition to functional impairments [1, 2], which are generally higher for patients who have failed ≥1 prior migraine preventive treatment [3–5]. Many patients with migraine either cannot tolerate the side effects, or do not respond to oral migraine preventive medications [6, 7]. As such, adherence to treatment is poor and the rate of patients discontinuing preventive therapy is high, especially among patients with chronic migraine (CM) [6, 7]. Given the poor adherence, efficacy, and tolerability, as well as the high rate of treatment discontinuations, patients with prior inadequate responses to multiple classes of migraine preventive medications are particularly in need of effective and tolerable long-term treatments for migraine prevention [8].

Calcitonin gene-related peptide (CGRP) is known to play a major role in migraine pathophysiology [9]. Biologic therapies targeting the CGRP pathway are the first preventive treatments for migraine that have been designed specifically to target the underlying pathophysiology of migraine [10]. Fremanezumab, a fully humanized monoclonal antibody (IgG2Δa), selectively targets α-CGRP and β-CGRP and is approved as a migraine preventive treatment in adults [11–13]. In previous double-blind (DB), placebo-controlled trials, fremanezumab demonstrated efficacy, with favorable safety and tolerability in both episodic migraine (EM) and CM patients [14–18]. In the two 12-week phase 3 HALO EM and HALO CM trials, fremanezumab significantly reduced the monthly average number of migraine days and the monthly number of headache days of at least moderate severity when compared with patients receiving placebo [14, 15]. In the HALO long-term safety study, both fremanezumab quarterly and fremanezumab monthly were well tolerated and demonstrated sustained improvements in monthly migraine days, headache days, and headache-related disability for up to 12 months in patients with migraine [19].

In the 12-week, randomized, DB period of the phase 3b FOCUS trial (ClinicalTrials.gov identifier: NCT03308968), fremanezumab demonstrated efficacy and tolerability as a quarterly or monthly migraine preventive treatment in adults with EM or CM and documented prior inadequate response to 2 to 4 migraine preventive medication classes [18]. The objective of the open-label extension (OLE) of the FOCUS study was to further evaluate the long-term efficacy, safety, and tolerability of monthly and quarterly fremanezumab.

## Methods

### Study design and participants

**Fig. 1:**
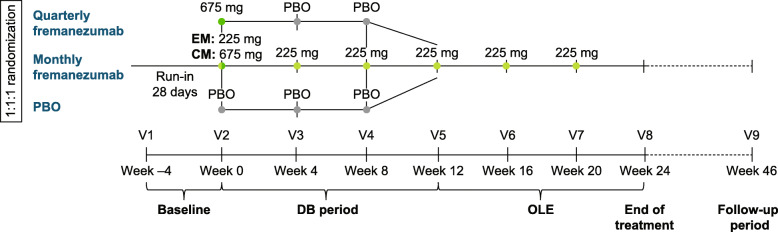
FOCUS study design. PBO, placebo; EM, episodic migraine; CM, chronic migraine; V, visit; DB, double-blind; OLE, open-label extension

The FOCUS study was an international, multicenter, randomized, phase 3b trial consisting of a 12-week, DB, placebo-controlled treatment period and a 12-week OLE, with a final follow-up 6 months after the last dose of fremanezumab (Fig. 1). The FOCUS study has been described in detail in previous reports [18]; as such, key details are summarized below. Participants in the FOCUS study were adults (18–70 years), with a diagnosis of EM or CM at or before 50 years of age for ≥12 months prior to the screening visit. Participants with EM had a headache on ≥6 days, but < 15 days, per month with ≥4 days fulfilling criteria from the International Classification of Headache Disorders 3 beta version (ICHD-3 beta) for migraine, probable migraine, or use of triptans or ergot derivatives to treat an established headache. Participants with CM had a headache on ≥15 days per month, with ≥8 days fulfilling the ICHD-3 beta criteria for migraine, probable migraine, or use of triptans or ergot derivatives to treat an established headache [20].

At the screening visit, study participants were required to have had a documented (in medical chart or by treating physician’s confirmation) prior inadequate response to 2 to 4 classes of migraine preventive medications within the past 10 years: angiotensin II receptor antagonists, anticonvulsants, β-blockers, calcium channel blockers, tricyclic antidepressants, onabotulinumtoxinA, or valproic acid. Inadequate response was defined as a lack of efficacy, poor tolerability, or treatment contraindicated/unsuitable for migraine prevention for the patient. For the DB period, eligible patients were randomized (1:1:1) to receive placebo or subcutaneously administered fremanezumab quarterly (675 mg/placebo/placebo) or monthly (EM: 225 mg/225 mg/225 mg; CM: 675 mg/225 mg/225 mg). All patients who completed the DB period were eligible to enter the nonrandomized, 12-week OLE and receive 3 monthly doses (225 mg) of fremanezumab (Fig. 1).

### Outcomes

Results from the DB period and the OLE were stratified according to randomization group for the DB period. During the DB period and the OLE, for the following outcomes, efficacy was measured as the mean change from baseline (assessed during the 28-day baseline period before the first DB dose) during the 12 weeks after the first dose of the DB period and the OLE: monthly average number of migraine days, average monthly headache days of at least moderate severity, days of acute headache medication use, days with photophobia/phonophobia, and days with nausea/vomiting. Efficacy was also measured according to the proportion of patients achieving ≥50% and ≥75% reduction in the monthly average number of migraine days in the 12 weeks after the first dose of the DB period and the OLE and as the mean change in disability from baseline through the 4 weeks after the last dose of study drug in the DB period and the OLE. Disability was evaluated by the Migraine Disability Assessment (MIDAS) [21] and the 6-item Headache Impact Test (HIT-6) [22]. Safety and tolerability were measured by the rates of adverse events (AEs), serious AEs (SAEs), and AEs leading to study discontinuation.

### Statistical analysis

The safety analysis set comprised all randomly assigned participants who received ≥1 dose of study drug. Participants in the intent-to-treat analysis set who received ≥1 dose of study drug and had ≥10 days of postbaseline efficacy assessments for the primary outcome (modified intent-to-treat analysis set) were included in all efficacy analyses. Demographics, baseline characteristics, efficacy, and safety outcomes in each treatment group were summarized descriptively.

## Results

### Patients

Of the 838 patients randomized for the DB period, 807 (96%) entered the OLE (264, placebo group; 271, DB quarterly fremanezumab group; 272, DB monthly fremanezumab group). Of the patients entering the OLE, 772 (96%) completed the OLE (253, placebo group; 259, DB quarterly fremanezumab group; 260, DB monthly fremanezumab group); 92% of patients completed both the full 6 months of DB and OLE treatment. Overall, 35 (4%) patients discontinued treatment in the OLE, including 17 (2%) due to withdrawal of consent, 6 (<1%) due to AEs, 3 (<1%) due to lack of efficacy, 2 (<1%) each due to protocol deviations and lost to follow-up, 1 (<1%) due to noncompliance with study procedures, and 4 (<1%) due to other reasons.

Among the patients in the OLE, baseline characteristics were similar across treatment groups and resembled those in the DB treatment period (Table 1). The mean (standard deviation [SD]) age was 46.4 (11.0) years, and patients ranged from 18 to 71 years of age; most patients were female (84%) and White (94%). The mean (SD) time since migraine diagnosis was 24.3 (13.3) years. More patients had CM (61%) than EM (39%), and 398 (49%) patients had a prior inadequate response to 2 migraine preventive medications.

**Table 1:** Demographic and Baseline Characteristics According to DB Randomization (OLE Safety Analysis Set)

	Placebo^**a**^(***n*** = 262)	Quarterly fremanezumab^**a**^(***n*** = 271)	Monthly fremanezumab^**a**^(***n*** = 274)	Total(***n*** = 807)
Age, mean (SD), years	46.9 (11.2)	46.0 (11.0)	46.1 (11.0)	46.4 (11.0)
Female sex, n (%)	218 (83)	226 (83)	230 (84)	674 (84)
Race, n (%)				
White	247 (94)	258 (95)	254 (93)	759 (94)
Black/African American	1 (<1)	1 (<1)	4 (1)	6 (<1)
Asian	1 (<1)	0	2 (<1)	3 (<1)
American Indian or Alaska Native	0	0	1 (<1)	1 (<1)
Other	1 (<1)	2 (<1)	1 (<1)	4 (<1)
Not reported	12 (5)	10 (4)	12 (4)	34 (4)
Weight, mean (SD), kg	71.3 (13.9)	70.5 (13.3)	71.1 (13.8)	71.0 (13.7)
Height, mean (SD), cm	167.6 (9.0)	167.6 (7.9)	167.4 (7.6)	167.6 (8.2)
Body mass index, mean (SD), kg/m^2^	25.3 (4.1)	25.0 (4.1)	25.3 (4.4)	25.2 (4.2)
Years since initial migraine diagnosis, mean (SD)	24.3 (13.4)	24.4 (12.9)	24.3 (13.7)	24.3 (13.3)
Migraine classification, n (%)				
Episodic migraine	105 (40)	102 (38)	106 (39)	313 (39)
Chronic migraine	157 (60)	169 (62)	168 (61)	494 (61)
Number of prior preventive medications failed, n (%)				
2	131 (50)	138 (51)	129 (47)	398 (49)
3	77 (29)	82 (30)	94 (34)	253 (31)
4	54 (21)	49 (18)	49 (18)	152 (19)
Monthly average number of migraine days, mean (SD)^b^	14.4 (6.2)	14.2 (5.6)	14.0 (5.5)	14.2 (5.8)
Headache days of at least moderate severity, mean (SD)^b^	12.9 (5.9)	12.5 (5.8)	12.6 (5.7)	12.7 (5.8)
Days per month of acute headache medication use, mean (SD)^b^	12.4 (6.3)	12.9 (6.2)	12.1 (5.9)	12.5 (6.1)
Days per month with photophobia/phonophobia, mean (SD)^b^	9.9 (7.8)	9.5 (6.8)	9.4 (6.8)	9.6 (7.2)
Days per month with nausea/vomiting, mean (SD)^b^	6.4 (6.0)	6.7 (5.9)	6.6 (5.9)	6.5 (5.9)
HIT-6 score, mean (SD)^b^	64.1 (4.8)	64.3 (4.3)	63.9 (4.5)	64.1 (4.5)
MIDAS score, mean (SD)^b^	62.0 (57.4)	62.2 (49.3)	61.8 (51.3)	62.0 (50.6)

*DB* double-blind, *OLE* open-label extension, *SD* standard deviation, *HIT-6* 6-item Headache Impact Test, *MIDAS* Migraine Disability Assessment, *mITT* modified intent-to-treat

^a^All patients in the OLE received fremanezumab 225 mg monthly

^b^OLE mITT analysis set

At baseline, for the DB placebo, DB quarterly fremanezumab, and DB monthly fremanezumab groups, the mean (SD) monthly average number of migraine days was 14.4 (6.2), 14.2 (5.6), and 14.0 (5.5), respectively, and mean (SD) headache days of at least moderate severity was 12.9 (5.9), 12.5 (5.8), and 12.6 (5.7), respectively. At baseline, for the DB placebo, DB quarterly fremanezumab, and DB monthly fremanezumab groups, the mean (SD) days per month of acute medication use was 12.4 (6.3), 12.9 (6.2), and 12.1 (5.9), respectively. At baseline, for the DB placebo, DB quarterly fremanezumab, and DB monthly fremanezumab groups, mean (SD) days per month with photophobia/phonophobia was 9.9 (7.8), 9.5 (6.8), and 9.4 (6.8), respectively, and mean (SD) days per month with nausea/vomiting was 6.4 (6.0), 6.7 (5.9), and 6.6 (5.9), respectively. At baseline, for the DB placebo, DB quarterly fremanezumab, and DB monthly fremanezumab groups, the mean (SD) HIT-6 score was 64.1 (4.8) points, 64.3 (4.3) points, and 63.9 (4.5) points, respectively, and mean (SD) MIDAS score was 62.0 (57.4) points, 62.2 (49.3) points, and 61.8 (51.3) points, respectively.

### Efficacy

**Fig. 2:**
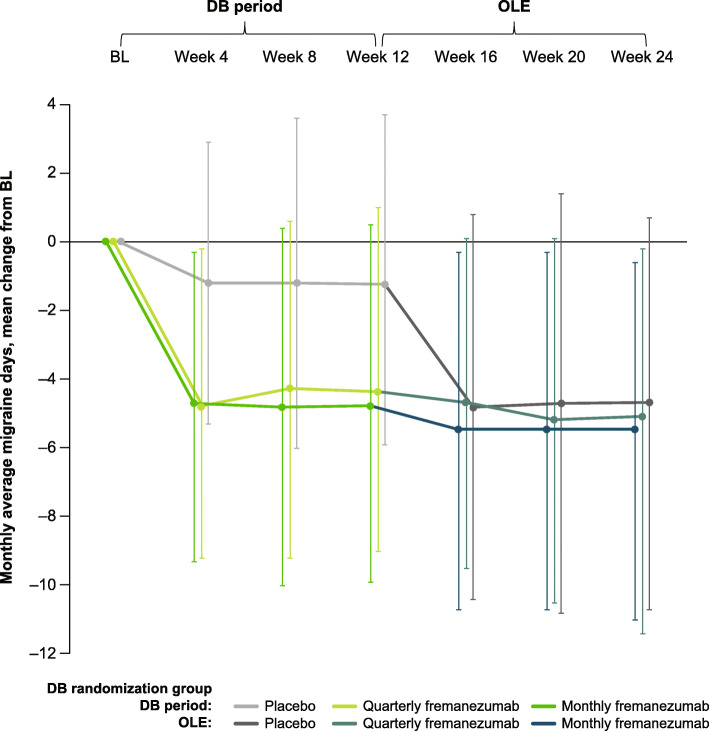
Mean change from BL in the monthly average number of migraine days over 6 months (mITT).^a ^BL, baseline; mITT, modified intent-to-treat; DB, double-blind; OLE, open-label extension. ^a^All patients in the OLE received fremanezumab 225 mg monthly

Over the 12-week DB period, the mean (SD) change from baseline in the monthly average number of migraine days was: placebo, − 1.2 (4.0); quarterly fremanezumab, − 4.4 (4.2); and monthly fremanezumab, − 4.8 (4.4). Over the 12-week OLE, patients had fewer monthly average migraine days (mean [SD] change from baseline: DB placebo, − 4.7 [5.4]; DB quarterly fremanezumab, − 5.1 [4.7]; DB monthly fremanezumab, − 5.5 [5.0]; Fig. 2).

**Fig. 3:**
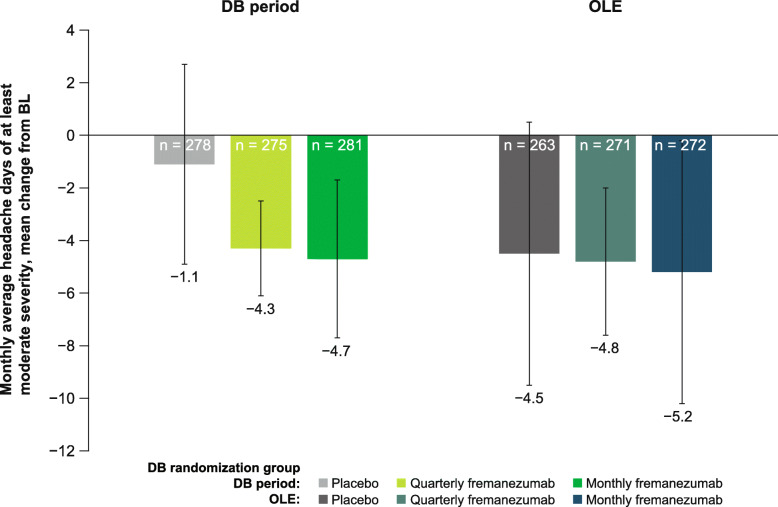
Mean change from BL in the number of headache days of at least moderate severity in the DB period and the OLE (mITT).^a^ BL, baseline; DB, double-blind; OLE, open-label extension; mITT, modified intent-to-treat. ^a^All patients in the OLE received fremanezumab 225 mg monthly

Over the 12-week DB period, the mean (SD) change from baseline in monthly headache days of at least moderate severity was: placebo, − 1.1 (3.8); quarterly fremanezumab, − 4.3 (4.1); and monthly fremanezumab, − 4.7 (4.6). Over the 12-week OLE, patients also had fewer monthly headache days of at least moderate severity (mean [SD] change from baseline: placebo, − 4.5 [5.0]; DB quarterly fremanezumab, − 4.8 [4.5]; DB monthly fremanezumab, − 5.2 [4.9]; Fig. 3).

**Fig. 4:**
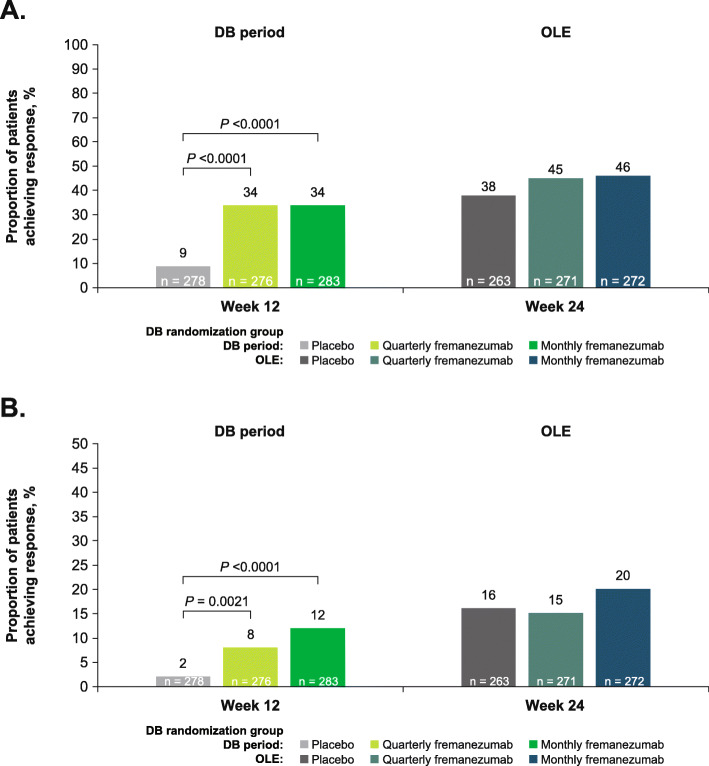
Proportion of patients achieving **a** ≥50% reduction and **b** ≥75% reduction in the monthly average number of migraine days in the DB period and the OLE (mITT).^a^ DB, double-blind; OLE, open-label extension; mITT, modified intent-to-treat. ^a^All patients in the OLE received fremanezumab 225 mg monthly

**Fig. 5:**
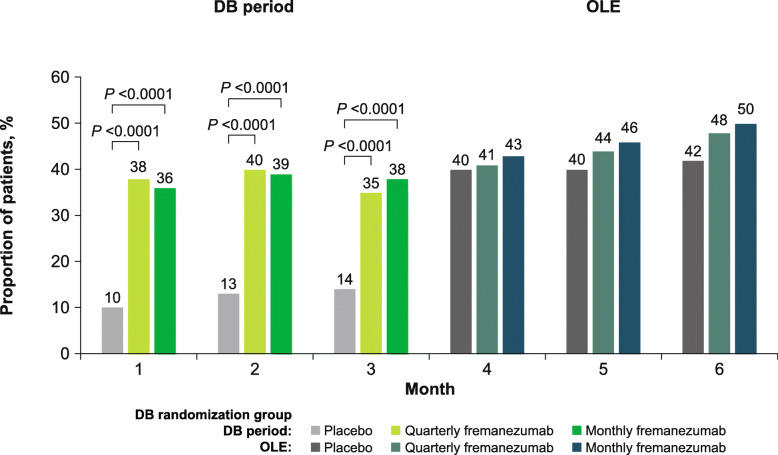
Proportion of patients achieving ≥50% reduction in the monthly average number of migraine days over 6 months (mITT).^a^ mITT, modified intent-to-treat; DB, double-blind; OLE, open-label extension. ^a^All patients in the OLE received fremanezumab 225 mg monthly

At 12 weeks of treatment in the DB period, 9% and 2% of patients in the placebo group achieved ≥50% and ≥ 75% reductions in the monthly average number of migraine days, respectively, compared with 34% and 8% in the quarterly fremanezumab group, and 34% and 12% in the monthly fremanezumab group. At 24 weeks, similar proportions of patients achieved ≥50% and ≥ 75% reductions in the monthly average number of migraine days across the placebo (38% and 16%), DB quarterly fremanezumab (45% and 15%), and DB monthly fremanezumab (46% and 20%) treatment groups (Fig. 4a and b). In addition, responder rates generally increased over time (Fig. 5).

**Fig. 6:**
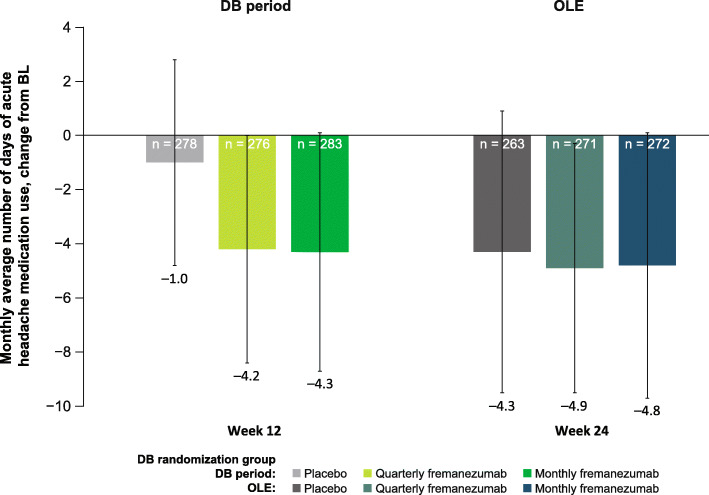
Mean change from BL in days of acute headache medication use in the DB period and the OLE (mITT).^a ^BL, baseline; DB, double-blind; OLE, open-label extension; mITT, modified intent-to-treat. ^a^All patients in the OLE received fremanezumab 225 mg monthly

Over the 12-week DB period, the mean (SD) change from baseline in days per month of acute headache medication use was: placebo, − 1.0 (3.8); quarterly fremanezumab, − 4.2 (4.2); and monthly fremanezumab, − 4.3 (4.4). Over the 12-week OLE, patients had fewer days per month of acute headache medication use (mean [SD] change from baseline: placebo, − 4.3 [5.2]; DB quarterly fremanezumab, − 4.9 [4.6]; DB monthly fremanezumab, − 4.8 [4.9]; Fig. 6).

**Fig. 7:**
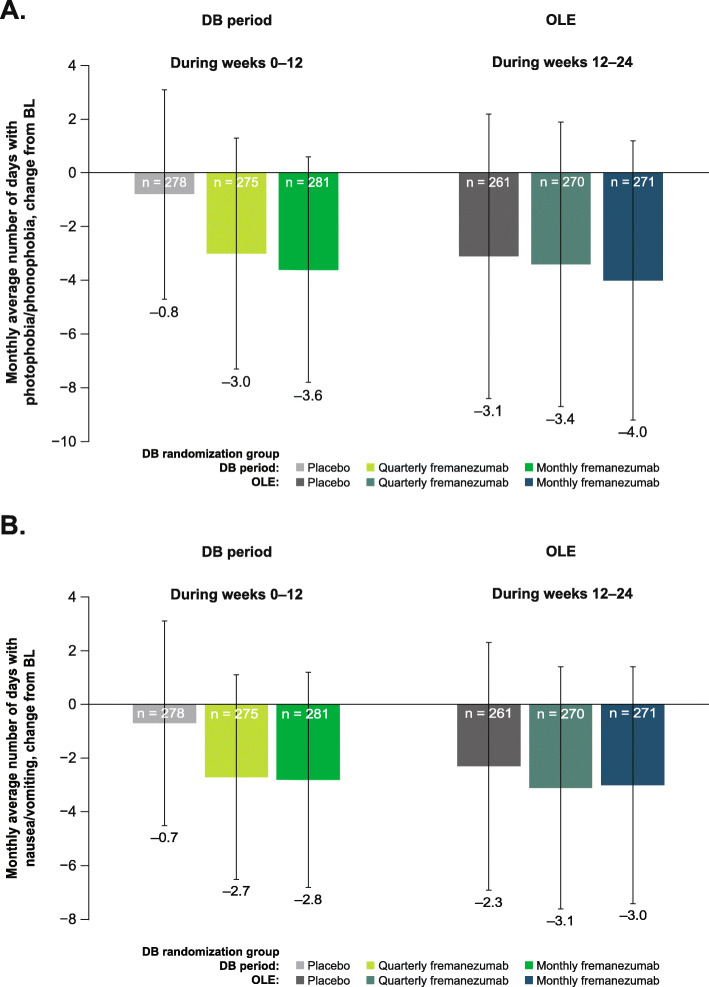
Mean change from BL in days with **a** photophobia/phonophobia and **b** nausea/vomiting in the DB period and the OLE (mITT).^a^ BL, baseline; DB, double-blind; OLE, open-label extension; mITT, modified intent-to-treat. ^a^All patients in the OLE received fremanezumab 225 mg monthly

Over the 12-week DB period, the mean (SD) change from baseline in days per month with photophobia/phonophobia was: placebo, − 0.8 (3.9); quarterly fremanezumab, − 3.0 (4.3); and monthly fremanezumab, − 3.6 (4.2). The mean (SD) change from baseline in the monthly number of days with nausea and vomiting was: placebo, − 0.7 (3.8); quarterly fremanezumab, − 2.7 (3.8); and monthly fremanezumab, − 2.8 (4.0). Over the 12-week OLE, patients reported fewer days per month with photophobia/phonophobia (mean [SD] change from baseline: placebo, − 3.1 [5.3]; DB quarterly fremanezumab, − 3.4 [5.3]; DB monthly fremanezumab, − 4.0 [5.2]; Fig. 7a) and fewer days per month with nausea or vomiting (mean [SD] change from baseline: placebo, − 2.3 [4.6]; DB quarterly fremanezumab, − 3.1 [4.5]; DB monthly fremanezumab, − 3.0 [4.4]; Fig. 7b).

**Fig. 8:**
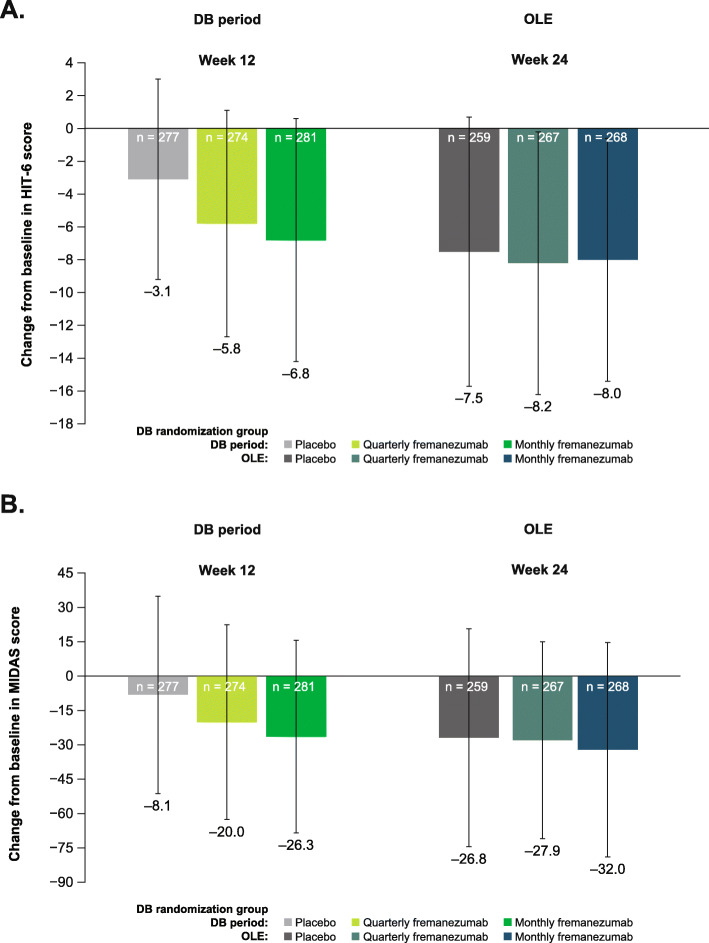
Mean change in disability in the DB period and the OLE as measured by **a** HIT-6 and **b** MIDAS (mITT).^a,b^ BL, baseline; DB, double-blind; OLE, open-label extension; HIT-6, Headache Impact Test; MIDAS, Migraine Disability Assessment; mITT, modified intent-to-treat. ^a^All patients in the OLE received fremanezumab 225 mg monthly

Over the 12-week DB period, the mean (SD) change from baseline in HIT-6 score was: placebo, − 3.1 (6.1); quarterly fremanezumab, − 5.8 (6.9); and monthly fremanezumab, − 6.8 (7.4). The mean (SD) change from baseline in MIDAS score was: placebo, − 8.1 (43.1); quarterly fremanezumab, − 20.0 (42.5); and monthly fremanezumab, − 26.3 (42.0). Over the 12-week OLE, patients had reductions in disability scores as measured by HIT-6 (mean [SD] change from baseline: placebo, − 7.5 [8.2]; DB quarterly fremanezumab, − 8.2 [8.0]; and DB monthly fremanezumab, − 8.0 [7.4]; Fig. 8a) and MIDAS (mean [SD] change from baseline: placebo, − 26.8 [47.6]; DB quarterly fremanezumab, − 27.9 [43.0]; and DB monthly fremanezumab, − 32.0 [46.8]; Fig. 8b).

### Safety and tolerability

During the 12-week DB period, the incidences of AEs (placebo, 48%; quarterly fremanezumab, 55%; monthly fremanezumab, 45%), SAEs (all groups, 1%), treatment-related AEs (placebo, 20%; quarterly fremanezumab, 21%; monthly fremanezumab, 19%), and protocol-defined AEs of special interest (placebo, <1%; quarterly fremanezumab, 1%; monthly fremanezumab, 1%) were similar across treatment groups (Table 2). During the 12-week OLE, the incidences of AEs (placebo, 52%; DB quarterly fremanezumab, 55%; DB monthly fremanezumab, 57%), SAEs (all groups, 3%), treatment-related AEs (placebo, 16%; DB quarterly fremanezumab, 17%; DB monthly fremanezumab, 20%), and protocol-defined AEs of special interest (placebo, 2%; DB quarterly fremanezumab, <1%; DB monthly fremanezumab, 3%) were similar across treatment groups. Cardiovascular AEs were infrequent and similar across treatment groups (≤1%) during both the 12-week DB period and the 12-week OLE (Table 2). The most common AEs reported during the OLE were nasopharyngitis (8%), injection site erythema (6%), injection site induration (5%), migraine (4%), and injection site pain (3%). During OLE, 4 patients had abnormal systolic blood pressure values (placebo, 1 [<1%]; DB quarterly fremanezumab, 2 [<1%]; DB monthly fremanezumab, 1 [<1%]) while 10 patients had abnormal diastolic blood pressure values (placebo, 4 [2%]; DB quarterly fremanezumab, 3 [1%]; DB monthly fremanezumab, 3 [1%]). During OLE, 10 patients reported constipation: 6 (2%) in DB placebo group, 2 (<1%) in DB quarterly fremanezumab group, and 2 (<1%) in DB monthly fremanezumab group. Overall, 7 patients discontinued the OLE due to AEs, including 4 (2%) patients in the DB placebo group, 1 (<1%) patient in the DB quarterly fremanezumab group, and 2 (<1%) patients in the DB monthly fremanezumab group. As with the DB period of the study, there were no deaths reported in the OLE or the follow-up period of the study.

**Table 2:** AEs in the DB Period and the OLE (Safety Analysis Set)

	Placebo		Quarterly fremanezumab		Monthly fremanezumab	
AE, n (%)	DB period (*n* = 277)	OLE^a^ (*n* = 262)	DB period (*n* = 276)	OLE^a^ (*n* = 271)	DB period (*n* = 285)	OLE^a^ (*n* = 274)
Any AE	134 (48)	137 (52)	151 (55)	149 (55)	129 (45)	155 (57)
Any SAE^b, c^	4 (1)	9 (3)	2 (< 1)	7 (3)	4 (1)	7 (3)
Treatment-related AE	55 (20)	41 (16)	57 (21)	47 (17)	55 (19)	56 (20)
Protocol-defined AE of special interest^d^	2 (<1)	4 (2)	3 (1)	2 (<1)	3 (1)	9 (3)
Death	0	0	0	0	0	0
AE leading to discontinuation^e, f^	3 (1)	4 (2)	1 (<1)	1 (<1)	4 (1)	2 (<1)
Cardiovascular AEs	3 (1)	3 (1)	2 (<1)	1 (<1)	4 (1)	4 (1)
Extrasystoles	0	2 (<1)	0	0	0	0
Palpitations	2 (<1)	1 (<1)	1 (<1)	0	2 (<1)	1 (<1)
Atrial fibrillation	0	0	0	1 (<1)	1 (<1)	0
Supraventricular tachycardia	0	0	1 (<1)	0	0	0
Tachycardia	0	1 (<1)	0	0	1 (<1)	1 (<1)
Bradycardia	1 (<1)	0	0	0	0	0
Left bundle branch block	0	0	0	0	0	1 (<1)
Coronary artery disease	0	0	0	0	0	1 (<1)^g^

*AE* adverse event, *DB* double-blind, *OLE* open-label extension, *SAE* serious adverse event, *AST* aspartate aminotransferase, *ALT* alanine aminotransferase, *ULN* upper limit of normal, *INR* international normalized ratio

^a^All patients in the OLE received fremanezumab 225 mg monthly

^b^DB period: thoracic vertebral fracture, uterine leiomyoma, vulval cancer, hypoesthesia, and metrorrhagia in the placebo group; atrial fibrillation, cholelithiasis, clavicle fracture, foot fracture, respiratory fume inhalation, rib fracture, road traffic accident, back pain, nephrolithiasis, and vocal cord thickening in the fremanezumab groups

^c^OLE: retinal tear, anal polyp, acute cholecystitis, cholelithiasis, anaphylactic reaction, diverticulitis, abnormal INR, angiomyxoma, intracranial aneurysm, multiple sclerosis, optic neuritis, nephrolithiasis, renal colic, dysmenorrhea, endometriosis, menometrorrhagia, and menorrhagia in the fremanezumab groups

^d^Ophthalmic-related AEs of at least moderate severity, events of possible drug-induced liver injury (AST or ALT ≥3 ULN, total bilirubin ≥2 ULN or INR >1.5), Hy’s law events, or events of anaphylaxis and severe hypersensitivity reactions

^e^DB period: chest discomfort, injection-site pain, and vulval cancer in the placebo group; palpitations, fatigue, cholelithiasis, road traffic accidents, and temporal arteritis in the fremanezumab groups

^f^OLE: upper abdominal pain, nausea, injection-site reactions, breast cancer, dizziness, headache, oropharyngeal pain, and hyperhidrosis in the placebo group; injection-site reactions, depressed mood, and asthma in the fremanezumab groups

^g^Patient experienced a non-serious event of coronary artery disease on day 211 of the study. The event was considered not related to study treatment by the investigator and was considered likely due to chronic pre-existing disease. The event was ongoing at the time of the last visit

## Discussion

Results from this 12-week OLE of the FOCUS study demonstrate that treatment with fremanezumab can lead to substantial clinical benefit in patients with EM or CM who previously did not respond to up to 4 different classes of migraine preventive medications; furthermore, in combination with the results from the 12-week DB period [18], these data indicate sustained clinical benefit with fremanezumab for up to 6 months in these patients.

Migraine is associated with a significant burden of disease, and the burden is greater for patients who have failed prior treatments [3]. Furthermore, estimates suggest that a large proportion of patients with migraine have failed ≥1 prior migraine preventive treatment [3]. Additionally, most health technology assessment bodies only recommend reimbursements for monoclonal antibody migraine preventive medication treatments for patients who failed prior treatments. In the 12-week, phase 3, double-blind CONQUER trial of patients with 2 to 4 prior migraine preventive medication failures, patients receiving galcanezumab had 4.1 fewer monthly migraine days compared with 1.0 fewer day among patients receiving placebo [23]. In a 12-week, phase 2 trial of patients with CM and ≥2 prior preventive treatment failures, patients receiving erenumab 70 mg and erenumab 140 mg had 2.7 and 4.3 fewer monthly migraine days, respectively, when compared to placebo [24]. The 12-week, phase 3, double-blind LIBERTY study evaluated erenumab among adults with EM and prior unsuccessful treatment with 2 to 4 migraine preventive treatments, and patients receiving erenumab had 1.6 fewer monthly migraine days when compared to placebo [25]. In the current study, over the 12-week DB period, patients in the quarterly fremanezumab and monthly fremanezumab treatment groups had 4.4 and 4.8 fewer monthly average migraine days, respectively, when compared to baseline. Furthermore, improvements in monthly migraine days were sustained over 24 weeks of fremanezumab treatment, with patients in the DB quarterly fremanezumab and DB monthly fremanezumab treatment groups showing 5.1 and 5.5 fewer monthly migraine days, respectively, when compared to baseline. Similarly, with fremanezumab treatment, improvements across all efficacy outcomes were not only sustained but showed continued improvement up to 6 months. The responder rates generally increased over time, suggesting that perhaps patients can achieve greater clinical benefit over time. In addition, there was no evidence of tachyphylaxis during this study period.

Of the 838 patients randomized during the DB period, 807 (96%) entered the OLE. Of the patients entering the OLE, 772 (96%) completed the OLE. During both the DB period and the OLE, few patients discontinued the study due to AEs, with most discontinuations due to withdrawal of consent or protocol deviations. In clinical practice, adverse effects are a major reason for discontinuation of many migraine preventive medications. Results from the FOCUS study demonstrated a low rate of treatment-emergent AEs and discontinuations due to AEs. Rates of AEs, SAEs, treatment-related AEs, and protocol-defined AEs of special interest were similar between the DB period and the OLE. The favorable cardiovascular safety profile observed in the OLE was similar to the findings from the phase 2b/3 fremanezumab studies [26]. No safety signals were identified during the study. This demonstrates that fremanezumab is safe and well tolerated in this difficult-to-treat population. In addition, low rates of treatment persistence may occur with traditional migraine preventive medications, with observational studies showing persistence ranging from 19% to 79% at 6 months and 7% to 55% at 12 months [27]. The low percentage of patients who discontinued this study due to AEs demonstrates that fremanezumab was well tolerated, a finding that is further supported by the low rates of treatment-emergent AEs and SAEs observed during the study.

This study had a few limitations. The OLE was uncontrolled with no placebo group or an active comparator. In addition, only patients who received treatment benefit and completed the 12-week DB period participated in the OLE. Furthermore, longer-term treatment beyond 6 months has not been evaluated in this population.

## Conclusions

In this 6-month study, patients with EM or CM and prior documented inadequate responses to 2 to 4 migraine preventive treatment classes had fewer monthly average migraine days, fewer headache days of moderate severity, fewer days of acute headache medication use, fewer days with photophobia and phonophobia, fewer days of nausea or vomiting, and reduced HIT-6 and MIDAS scores. Improvements in these outcomes in the OLE were also of greater magnitude than the improvements seen at the end of the 3-month DB period. In conclusion, findings from this FOCUS study indicate that fremanezumab is effective, safe, and well tolerated for up to 6 months in patients who had previously not responded to 2 to 4 classes of migraine preventive medications.

## Data Availability

Anonymized data, as described in this manuscript, will be shared upon request from any qualified investigator by the author investigators or Teva Pharmaceutical Industries, Ltd.

## Abbreviations

CM: Chronic migraine
CGRP: Calcitonin gene-related peptide
DB: Double-blind
EM: Episodic migraine
OLE: Open-label extension
ICHD-3 beta: International Classification of Headache Disorders 3 beta version
MIDAS: Migraine Disability Assessment
HIT-6: 6-item Headache Impact Test
AE: Adverse event
SAE: Serious adverse events
SD: Standard deviation
